# Population pharmacokinetics and pharmacodynamics of two dosing regimens of antenatal corticosteroids: protocol for a prospective nested study in a randomised controlled trial

**DOI:** 10.1136/bmjopen-2024-096523

**Published:** 2025-06-08

**Authors:** The WHO ACTION-III PK PD Collaborators, Ebunoluwa A Adejuyigbe

**Keywords:** CLINICAL PHARMACOLOGY, OBSTETRICS, NEONATOLOGY

## Abstract

**Introduction:**

Antenatal corticosteroid (ACS) regimens have remained unchanged since the initial trials in 1972, with the optimal regimen still undetermined. The WHO ACTION (Antenatal CorticosTeroids for Improving Outcomes in preterm Newborns)-III trial is a three-arm individually randomised double-blind trial evaluating the efficacy and safety of two different ACS dosing regimens (currently used and lower-dose ACS regimens vs placebo) in women with a high probability of having a late preterm birth. This study protocol nested within this trial aims to evaluate the pharmacokinetics (PK) and pharmacodynamics (PD) effects of two different ACS dosing regimens in pregnant women in the late preterm period (34–36 weeks) to help inform an optimal dosing regimen.

**Methods and analysis:**

The study will be conducted in two of the five countries participating in the WHO ACTION-III trial—India (Delhi, Belagavi) and Nigeria (Ibadan and Ile-Ife). We will use a population PK approach using sparse sampling to study the PK effects of the two ACS regimens, that is, 6 mg dexamethasone phosphate (DEXp) or 2 mg betamethasone phosphate (BETp), administered intramuscularly every 12 hours for a maximum of four doses or till birth, whichever is earlier, compared with placebo. We will also ascertain the fetal–maternal ratio of DEXp and BETp at birth.

Maternal venous blood samples will be collected at 0, 1–4 hours, 8–12 hours after the first dose, and at 24–36 hours, 48–60 hours, 72–96 hours after the last dose, and immediately after birth, along with cord blood. Concentrations of DEXp and BETp will be measured at set time points using a validated liquid chromatography mass spectroscopy assay. PD parameters measured will include total and differential white blood cell count (by automated analysers using electrical impedance), plasma glucose (hexokinase method) and serum cortisol (using a validated electrochemiluminescence immunoassay), at predefined time points. PK models will be developed for each drug using non-linear mixed effects methods. Optimal dosing will be investigated using Monte Carlo simulations.

**Ethics and dissemination:**

The study has been approved by the WHO Ethics Review Committee and the site-specific ethics committees of the participating leading institutions. Written informed consent will be obtained from all participants. The study results will be published in a peer-reviewed journal and presented at scientific conferences.

**Trial registration number:**

ISRCTN11434567.

**STRENGTHS AND LIMITATIONS OF THIS STUDY**

- This is the first study designed to provide in vivo data on pharmacokinetics (PK) and pharmacodynamics (PD) parameters of two different antenatal corticosteroid (ACS) regimens (the dose currently recommended in early preterm period and a lower dose) among pregnant women in the late preterm period (34–36 weeks of gestation).
- The study will also ascertain the maternal–fetal ratios of ACS at birth with these two regimens.
- The study includes a diverse population of pregnant women from South Asia (India) and Africa (Nigeria).
- Genetic differences among women that could impact the PK or PD of antenatal steroids, as well as variations in placental metabolism that may influence cord blood levels, have not been considered.

## Introduction

 High quality evidence supports the use of antenatal corticosteroid (ACS) for early preterm births (less than 34 weeks gestation),^1 2^ but the recommendations and therefore, the use of ACS in the late preterm period (34–36 weeks gestation) varies globally, given lack of unequivocal benefit–risk balance in this population.^3^,^5^ When ACS is administered to the mother and crosses the placenta into the fetal circulation, all fetal organ systems are exposed to steroids. Recent studies suggest an increase in the short-term adverse effects like neonatal hypoglycaemia^6^ and increased risk of infection in newborns extending into the first year after birth.^7 8^ Longer-term follow-up studies suggest a potential association between steroid exposure in utero and neurocognitive delays or behavioural difficulties in children,^9 10^ as well as potential links with adverse long-term health outcomes.^11^ Despite the fact, ACS, which has multiple widespread biological effects, has not been evaluated for the optimal choice of steroid salt, dose or duration of treatment despite being in use for over 50 years.^12^ Differences in the steroid base, salt, total dose and the dosing interval result in varying levels and duration of steroid exposure for the fetus and thereby differing benefit–risk balances; yet dose-ranging trials to evaluate safety and identify an optimal dosing regimen have never been performed.

Recent animal studies suggest that lung maturational changes can be affected at much lower doses of ACS than those conventionally used in clinical practice,^13^ and while studies on systemic ACS use for different clinical conditions in adults suggest that the risk of adverse effects is proportional to the corticosteroids dose,^14^ there are no studies evaluating a dose–response relationship between different ACS dose regimens and newborn outcomes. The recent BETADOSE trial that evaluated the effects of a 50% reduction in betamethasone dose on neonatal outcomes did not evaluate the pharmacokinetics (PK) and pharmacodynamics (PD) of the different dose regimens.^15^ The PK and PD effects of various ACS regimens are complex, especially as these drugs are administered to pregnant women to treat their fetuses. This complexity arises from several factors, including maternal PK, placental drug transfer and metabolism and fetal metabolism. Few studies have evaluated the PK and PD effects of ACS in pregnant women and their newborns.^12^

The WHO is currently coordinating the ACTION-III trial, a three-arm individually randomised double-blind trial to evaluate the safety and efficacy of different ACS regimens, that is, the dose currently recommended in the early preterm period (dexamethasone phosphate (DEXp) 6 mg every 12 hourly for a maximum of four doses or till birth, whichever is earlier), and a lower dose (betamethasone phosphate (BETp) 2 mg every 12 hourly, for a maximum of four doses or till birth, whichever is earlier), compared with placebo, among pregnant women in late preterm period (34–36 weeks of gestation).^5^ This ongoing study presents a unique opportunity to evaluate the PK and PD of these different ACS regimens in pregnant women that can be used for future calibration and optimisation of ACS dosing for late preterm births.

The primary objectives of the PK-PD study, nested within the trial above, are: (1) to study the PK of two different ACS regimens: BETp four doses of 2 mg each, administered intramuscularly every 12 hours; and DEXp four doses of 6 mg each, administered intramuscularly every 12 hours in pregnant women in the late preterm period (34 to <37 weeks gestation); (2) to study PD effects (plasma glucose, total and differential white blood cell (WBC) counts and serum cortisol) of the above dosing regimens on pregnant women and (3) to compare the PD parameters in pregnant women receiving the above two regimens to those who do not receive either drug (ie, women receiving placebo). The secondary objectives are to assess the concentrations of ACS in cord blood at birth and determine the fetal-to-maternal ratio when each of the two different regimens is used. Additionally, we will study PD parameters in the cord blood of ACS-treated women and compare these with those obtained from placebo-treated women. Furthermore, these parameters will be compared between the African (Nigerian) and South Asian (Indian) pregnant women.

## Methods and analyses

### Study setting

The WHO ACTION-III trial is being conducted across seven sites in five countries—Bangladesh, India, Kenya, Nigeria and Pakistan. Of these countries, the PK-PD study will be conducted in two countries—two sites in India (Delhi, Belagavi) and two sites in Nigeria (Ibadan and Ile-Ife). These two countries are selected to ensure diverse representation of South Asian and West African pregnant women, given the possible differences in their characteristics such as body mass index (BMI) and pregnancy weight gain.^16^,^18^ The study hospitals in these settings also had the capacity to meet blood sample processing and storage requirements required for this study.

### Study design

**Figure 1:**
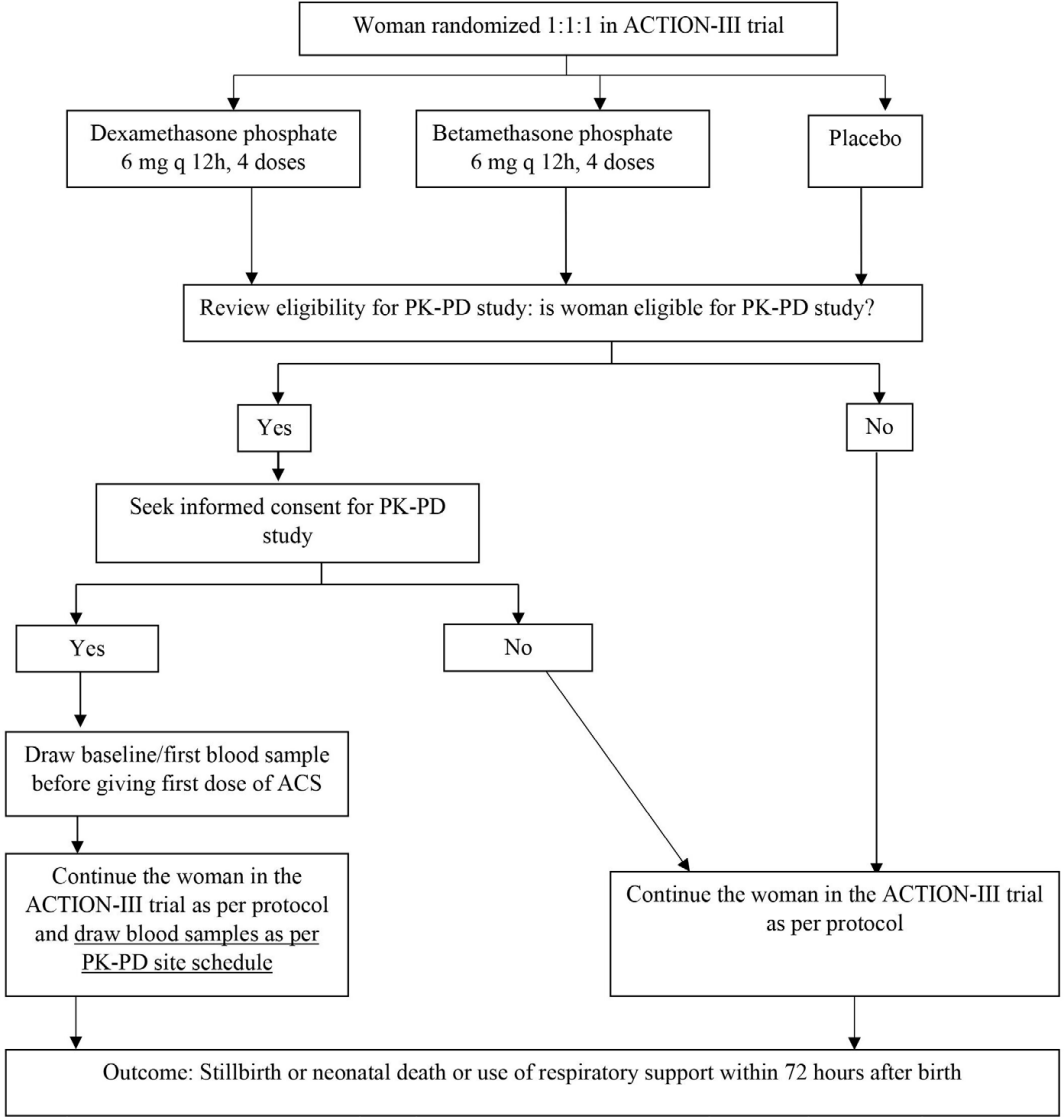
PK-PD study flow chart. ACTION, Antenatal CorticosTeroids for Improving Outcomes in preterm Newborns; ACS, antenatal corticosteroid; PD, pharmacodynamics; PK, pharmacokinetics.

The PK-PD study will be nested within the ACTION-III trial (figure 1). The ACTION-III trial enrols women with singleton or multiple pregnancy at 34 weeks 0 days to 36 weeks 5 days of gestation, with at least one live foetus, and a high probability of late preterm birth defined as birth expected between 12 hours and 7 days after randomisation. Women with clinical suspicion or evidence of clinical chorioamnionitis or severe infection, those having received any systemic corticosteroid outside of trial in the previous 2 weeks, no ultrasound assessment of gestational age, major or lethal congenital fetal anomaly, participating in another clinical trial, or having previously participated in any ACTION trial or having any contraindication to corticosteroids are excluded.^5^ Women consenting to participate in the ACTION-III trial are randomised in a 1:1:1 ratio to receive the investigational medicinal product (IMP), which could be any one of the three treatments as follows: (1) DEXp 6 mg intramuscularly 12 hourly for four doses (currently recommended dose) or (2) BETp 2 mg intramuscularly 12 hourly for four doses (lower dose) or (3) placebo. A single course of IMP is administered every 12 hours, to a total of four doses or until birth occurs, whichever occurs first. Enrolment into the PK-PD study: all women randomised into the ACTION-III trial at the selected participating hospitals will be assessed for eligibility for the PK-PD study, and if eligible (criteria listed below), will be enrolled in the PK-PD study following a written, informed consent.

### Population PK approach with sparse sampling

The study will use a population PK approach to describe the PK of BETp and DEXp^19^,^21^ in pregnant women in the late preterm period. Population PK modelling approaches rely on data from multiple individuals and often use pooled data from multiple patients, sampled at pragmatic intervals (sparse sampling). This approach is generally used to analyse clinical data collected in a setting where intense sampling (which requires multiple blood draws to obtain a full concentration/response time plot for every individual subject) is not practical. This sparse sampling approach has been used to study the PK of antiretrovirals in pregnancy, as well as in trials for paediatric medicines.^22^,^24^

### Study period

The study is expected to run over a period of 12 months, starting in the third/fourth quarter of 2024. Each enrolled participant will be part of the study for a maximum period of 132 hours after enrolment, beginning an hour prior to the first dose of IMP, up to 96 hours after the last dose. During this study period, blood samples for the measurement of PK and PD parameters from the woman will be drawn at prespecified time points described below. Cord blood will also be collected at birth.

### Study population

The inclusion criteria for the PK-PD study are aligned with those of the main ACTION-III trial, that is, pregnant women in the late preterm period (34–36 weeks gestation) who are eligible for and have been randomised in the ACTION-III trial (see online supplemental file 1, appendix panel 1 for ACTION-III trial eligibility criteria). The exclusion criteria for the PK-PD study are severe anaemia (haemoglobin (Hb) <7 mg/dL; based on the latest available Hb value from the third trimester to the current admission) or having received blood/blood product transfusion in 7 days prior to enrolment in the ACTION-III study.

Consecutive women enrolled and randomised into the ACTION-III trial will be approached during the study period and assessed for eligibility for the PK-PD study. Eligible women will be enrolled in the PK-PD study following written informed consent. The first (baseline) blood sample for the PK-PD study will be drawn before administering the first dose of IMP (DEXp or BETp or placebo) in the ACTION-III trial (figure 1).

### Sample size

In each country (India and Nigeria), complete samples from a minimum of 30 women who receive the active drug to enable association of covariates (country, maternal age (years) weight (kg), maternal height (cm), BMI, gestational age, fetal sex) with PK parameters will be required.^25^ Given that all consenting women may not be able to provide the required samples—due to an estimated 30%–40% delivering within 12 hours of the first ACS dose or withdrawing consent—the sample size for each group has been increased by 60% to account for these potential losses, that is, 50 women per arm (DEXp or BETp or placebo) in each country, that is, 150 women from India and 150 women from Nigerian sites.

### Study procedures

Serial maternal blood samples will be collected starting before the administration of the first dose of IMP up to 96 hours after the last dose, through a dedicated indwelling cannula or by venipuncture. A cord blood sample will also be collected at birth. The sampling schedule is provided in table 1. All study sites will collect the samples at time points within the sampling time window depicted in table 1 (see online supplemental appendix table 1 for site-specific schedules). The sampling time points for the PK and PD analysis are indicated in table 1.

**Table 1:** Blood sampling schedule

Sample number	Sampling time point	Maternal PK (BETp and DEXp)	Maternal PD (cortisol, glucose, WBC counts)	Cord PK+PD
1	*Baseline (−0.5 hours to 0 hours)*	√	√	
2	1–4 hours post first dose	√	√	
3	8–12 hours post first dose	√	√	
X*	At birth	√	–	√
4	24–36 hours after the last dose†	√	√	
5	48–60 hours after the last dose†		√	
6	72–96 hours after the last dose†		√	

PD parameters include serum cortisol, plasma glucose, total and differential WBC count (neutrophils, basophils and lymphocytes).

Windows for all draws ±30 min, except sample 6. Sample 6 to be drawn as close as possible to the stated time point for the site, otherwise at the point of discharge.

* Birth can happen at any time therefore, birth sample is not numbered sequentially and is labelled as X.

† Regardless of the time of birth.

BETp, betamethasone phosphate; DEXp, dexamethasone phosphate; PD, pharmacodynamics; PK, pharmacokinetics; WBC, white blood cell.

### Sample collection and processing

The blood samples at each time point will be collected in different vials depending on the analysis required: fluoride vial for glucose, plasma preparation tube (K2EDTA; dipotassium ethylenediaminetetraacetic acid) for WBC total and differential counts and steroid levels of DEX and BET, and serum separating tube for serum cortisol. The blood samples for glucose and WBC counts will be sent to the hospital-specific laboratories and analysed within 1 hour of collection while maintaining them at room temperature (below 25°C) during the entire time.

The serum and plasma sample aliquots for cortisol and steroid assays will be sent for analysis to a central study laboratory at Syngene International, Bangalore, India. For the cortisol assay, serum will be stored in a freezer (−20°C) immediately after aliquoting. For steroid assay, 200 µL of sodium arsenate solution will be added to the K2EDTA vacutainer tube kept in an ice bath, and the 4 mL blood sample will be added to this vacutainer tube. The tube will be gently inverted 8–10 times for thorough mixing and centrifuged at 3500 rpm for 10 min at 4°C, within 30 min from blood sample collection to separate the plasma. The plasma will be divided into two aliquots in cryovials with pre-added sodium fluoride solution (50 µL for 1 mL plasma), and immediately stored in the freezer (−20°C) until shipment.

All sites will ship the primary aliquots for cortisol and steroid samples to the central laboratory in India on dry ice under temperature-controlled conditions to ensure timely analysis of the samples within the 90-day time window for which the stability testing is proven for the analysis methods used. Quality checks will be undertaken at various steps, and back-up samples will be requested by the central study laboratory in case the primary samples cannot be analysed for any reason.

### Laboratory analysis-bioanalytical methods

For PD parameters, total and differential WBC counts will be measured using automated analysers that use the electrical impedance methodology.^26^ Plasma glucose will be measured using the hexokinase method.^27^ Serum cortisol will be measured by a validated electrochemiluminescence immunoassay. Plasma concentrations of DEX and BET will be measured using a validated liquid chromatography mass spectroscopy methodology for calculating PK parameters.

### Quality assurance

Standard operating procedures will be prepared for all study procedures including maternal and cord blood sample collection, processing, storage and handling. The site teams will undergo hands-on training in blood sample collection and processing procedures at the central study laboratory in India, followed by a dry run at each hospital under the supervision of the central study laboratory team and WHO trial coordinating unit (TCU). Procedural details regarding sample collection and processing, for example, sample collection to storage time, sample analysis time for glucose and WBC count, addition of various solutions during PK sample processing, storage temperature, etc, will be maintained in the form of logs at all sites hospitals and reviewed every 2–4 weeks by the WHO TCU. Regular site monitoring visits will be made by WHO TCU and independent, external monitors.

### Data collection and management

In addition to the main trial data that includes date and time of each dose of ACS and date and time of birth, additional data will be collected on the date and time of each blood sample (maternal and cord blood), estimated gestational age at time of first dose of ACS administration, time of the last dose, maternal characteristics including: age, body weight, height, BMI and liver and renal function if clinically indicated, and possible covariates such as time last meal taken, and concomitant medications.

Data will be managed centrally by a data management team, supervised directly by WHO project managers. The study statistician will be responsible for the development of the statistical analysis plan and reporting to the DSMB. A web-based, Good Clinical Practice (GCP) compliant data management platform (Kamolo, Centro Rosarino Estudios Perinatales) will be used and will be overseen by the site data managers. All data will be collected in study centres on paper case report forms. Quality control will be performed at each site, and a validation system will be built into the data entry and management system to ensure consistency, accuracy and completeness of the data collected.

### PK-PD data modelling and analysis

#### Data screening

The measured drug concentration versus data will be initially graphed to determine consistency with PK expectations (declining concentrations after dosing) and to look for outliers. The PD will be grouped according to drug or placebo dosing for exploratory assessment of generally expected differences from baseline measurements.

#### Software

PK data and drug response PD data collected from the study will be modelled using NONMEM 7.5 (ICON Pic) software. For statistical analysis, graphics 4.3.2 (The R Project Group, www.r-project.org) will be used.

#### Estimation method

The first-order conditional estimation method with the interaction option implemented in NONMEM 7.5 will be primarily considered. Alternatively, the importance sampling method will be used for parameter estimation if the primary method fails due to sparse data.

#### Model building procedures

A base model will first be developed for the pretreatment and placebo measurements, the PK of each drug, and each PD biomarker using the models established by Krzyzanski *et al*.^28^ Once adequate base models have been developed, the joint drug/PD models and covariate effect models will be built. To ensure reasonable initial estimates, we will use the results from the Krzyzanski study.

#### Fixed effects models

**Figure 2:**
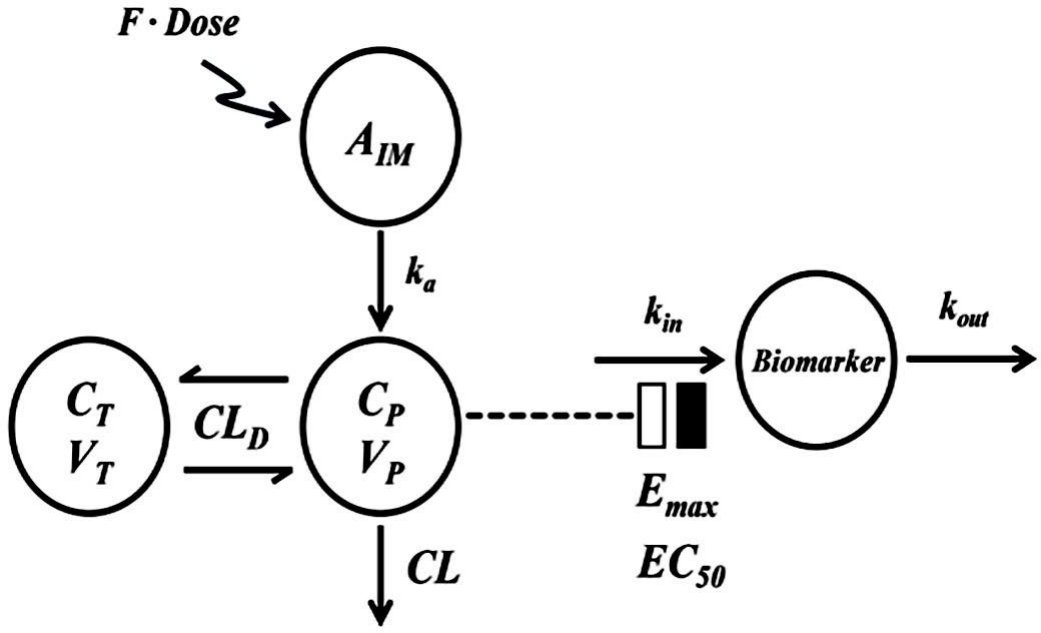
Schematic diagram of a PK-PD model describing DEX and BET plasma concentrations in pregnant women and their alteration of assessed biomarkers following multiple intramuscular dose administrations. BET, betamethasone; DEX, dexamethasone; PD, pharmacodynamics; PK, pharmacokinetics.

At stage 1, a two-compartment disposition model with first-order absorption will be used for describing plasma drug concentrations as the primary assessment (figure 2). Placebo cortisol data will be captured with the cosine circadian model. The inhibitory effect of the drugs on cortisol will be described by an indirect response model (figure 2).^29^ The cellular PD biomarkers will be described by inhibitory or stimulatory indirect response models that account for production (*k*_in_) and loss (*k*_out_) of the various biomarkers. Drug effects on plasma glucose will be described by a similar basic stimulatory indirect response model.

At stage 2, we will expand our meta-analysis of all available PK data for DEXp and BETp using a minimal physiologically based PK (mPBPK) model^30^ to compare the PK of these drugs in the current study. These drugs have linear PK that facilitates such comparison. An exploratory assessment will be made to determine whether it will be feasible to incorporate the designated covariates and consider the maternal PD in the mPBPK model.

#### Random effect models

The between-subject variability will be estimated for all parameters where feasible. A log-normal parameter variability model or other appropriate parameter variability models will be considered for the PK and PD parameters. For the residual unidentified variability, additive, proportional and a combined additive and proportional error models will be considered.

#### Covariate model

The subject population is a heterogeneous group of women but with limited age ranges. The broad protocol inclusion and exclusion criteria will create considerable inter-subject variability. Assessment of covariate effects such as race and body weights may be possible under these conditions.

#### Model performance diagnostics

Standard methods for diagnostics of model performance will be applied. These will include the value of the objective function, observed versus prediction plots, weighted residuals versus time and prediction plots and visual predictive checks.

#### Simulations

Simulations of model PK/PD variables will be performed to examine the data and model fittings in comparison to the expectations of previous PK/PD modelling of corticosteroids at higher doses in healthy and pregnant women.^28^,^30^

#### Statistical analysis

Drug-related PK and PD individual parameters (eg, V, CL, ka, Emax, EC50) will be summarised using descriptive statistics. Two-way analysis of variance will be applied to test for between drug differences. The population estimates of PK-PD parameters will be compared using 95% CIs. For the model selection, the χ^2^ test for significance of the change in the maximum likelihood objective function will be used. For the selection of significant covariates, the likelihood ratio test will be applied.

### Study oversight

In addition to the monitoring procedures for the ACTION-III trial in accordance with the International Council for Harmonisation of Technical Requirements for Pharmaceuticals for Human Use (ICH) Harmonised Tripartite Guideline for GCP E6 (R2), additional monitoring activities will be conducted at all PK-PD study hospitals by independent trial monitors, the central laboratory team (Syngene International) and WHO TCU. Day-to-day oversight will be done by the principal investigators and co-investigators.

### Patient and public involvement

Patients were not directly involved in the design of this study.

## Discussion

ACS therapy remains inadequately optimised. The minimal therapeutically effective dose to enable fetal lung maturity and to minimise adverse effects has not been ascertained despite 50 years of being in clinical use. This study will describe the PK and PD of two different regimens of ACS that include either DEX or BET phosphate, given in the currently recommended and lower dose regimens, respectively.

Research involving non-human primates and sheep suggests that fetal plasma concentrations should be kept above 1 ng/mL for a duration of 48 hours to promote fetal lung maturation while avoiding excessively high plasma levels.^12^ Although this is an estimated threshold based on studies in sheep and primates, similar conclusions have also been drawn from clinical data.^31^ We are aware of only three previous human studies that evaluated the PK and PD of DEX and BET in reproductive age but non-pregnant women,^32^ PK of BET in vivo in pregnant women^31^ and PK of DEX and BET ex vivo using placental perfusion.^33^ While the first study done in non-pregnant adult women showed that the currently administered ACS doses result in short-term PD effects like increased glucose, suppressed cortisol, increased neutrophils and suppressed basophils, CD3CD4 and CD3CD8 lymphocytes in women, the latter two suggest that lower doses of ACS can achieve the suggested optimal drug levels required for desirable effects in the fetus. The current study will be the first to provide these data for two different regimens of ACS, both currently recommended for the early preterm period and a lower dose, in pregnant women in late preterm gestation, alongside information on maternal-fetal transfer of ACS with these regimens.

Only one study, the BETADOSE trial, has evaluated a lower dose of ACS in pregnant women on neonatal outcomes until. This non-inferiority trial compared full (12 mg plus 12 mg, 24 hours apart) versus half (12 mg single dose) dose of BETp and acetate and could not show that half dose was non-inferior to full dose. However, the choice of half dose in this trial was pragmatic, based on its effectiveness in achieving similar lung maturation as the full dose in sheep, and the halving of the total dose of steroids was achieved by front-loading the entire drug exposure. Hence, questions remain whether non-inferiority could be achieved by distributing a halved dose over the normal duration of a standard treatment, which would be more consistent with recent and historical data suggesting that a low-amplitude, extended duration of fetal steroid exposure is important to achieve fetal lung maturation. The ACTION-III trial incorporates a low dose regimen that is based on PK modelling, which could potentially achieve lung maturation at least as effectively as the dose currently recommended for use in the early preterm period, that is, 6 mg DEXAp regimen, but with possibly less disruption to the hypothalamic–pituitary–adrenal axis.^34^

Our study uses a popPK modelling approach that relies on concentration-time data from multiple individuals and allows the analysis of clinical data collected in a setting where rich data are not practical, as in the high-risk population of pregnant women at risk of preterm birth in the ACTION-III trial. While it uses technically complex mathematical and compartmental methods to reach conclusions, it allows an integration of the covariate information (eg, weight, race/ethnicity, renal/hepatic function, concomitant medications, etc). Therefore, information provided by the popPK model can help optimise ACS dose regimens in this population. However, we will not be able to assess the impact of genetic differences among women that could affect the PK or PD of antenatal steroids, or variations in placental metabolism that may influence cord blood levels.

To conclude, complications from preterm birth continue to be the leading cause of neonatal mortality, particularly in low-resource settings. ACSs are currently the only known effective intervention to enhance survival rates and reduce morbidity among newborns. However, our understanding of the PK and PD of this life-saving treatment is limited. This study aims to shed light on the PK and PD of ACS when used in real-world settings. The data generated will contribute to optimising ACS regimens and improve our understanding of important issues related to dosing strategies and the effects of the drugs at a biological level.

### Ethics and dissemination

The study has been approved by the WHO Ethics Review Committee (20, Avenue Appia, Ch-1211, Geneva 27, Switzerland; ercsec@who.int), ref: 0003488) on 3 March 2021 and the site-specific ethics committees of the participating leading institutions (India—Institutional Ethics Committee, Vardhman Mahavir Medical College and Safdarjung Hospital, Delhi (Ref No IEC/VMMC/SJH/Project 2023-11/392) and Institutional Ethics Committee, Translational Health Science and Technology Institute, Haryana (Ref No: THS 1.8.1/(170); India—Institutional Ethics Committee of KLE Academy of Higher Education and Research, Belagavi (Ref No: KAHER/EC/2023–24/D-19062302); Nigeria—University of Ibadan and University College Hospital Ethics committee, Ibadan (Ref No UI/EC/23/0429); Nigeria—Ethics and Research Committee, Obafemi Awolowo University Teaching Hospitals Complex, Ile-Ife (ERC/2023/11/17; dated 30/11/2023)). A written informed consent will be taken from all participants in line with ICH GCP guidance.

The results of the study will be published in a peer-reviewed journal and presented at scientific conferences.

## Supplementary material

10.1136/bmjopen-2024-096523 online supplemental file 1
