# Supplementary material for: Population pharmacokinetics and pharmacodynamics of two dosing regimens of antenatal corticosteroids: protocol for a prospective nested study in a randomised controlled trial
Source: BMJ Open. 2025 Jun 8;15(6):e096523. doi: 10.1136/bmjopen-2024-096523 (PMC12161439; doi:10.1136/bmjopen-2024-096523)
Supplement: online supplemental file 1 [file bmjopen-15-6-s001.docx]

**SUPPLEMENTARY APPENDIX**

**Population pharmacokinetics and pharmacodynamics of two dosing regimens of antenatal corticosteroids: protocol for a prospective nested study in WHO ACTION-III randomized controlled trial.**

**Panel 1.** ACTION-III trial eligibility criteria

| **Inclusion criteria.**  Women with singleton or multiple pregnancy at 34 weeks 0 days to 36 weeks 5 days, with at least one live fetus, and a high probability of late preterm birth will be included. High probability of late preterm birth (up to 36 weeks 6 days) is defined as birth expected between 12 hours and 7 days after randomization as a result of one of the following:   1. preterm labour with intact membranes, where preterm labour is defined as at least 6 regular contractions/hour and at least one of the following: cervix ≥3 cm dilated or 75% effaced; or 2. membranes rupture without preterm labour (preterm labor defined as above; or 3. planned delivery by induction of labour or caesarean section between 24 hours and 7 days, as deemed necessary by the provider. An induction must be scheduled to start by 36 weeks 5 days at the latest, whereas a caesarean section must be scheduled by 36 weeks 6 days at the latest.   **Exclusion criteria.**  A woman is ineligible if:   - she is expected to give birth in <12 hours (i.e., if she had ruptured membranes with cervix dilated ≥3 cm or effaced ≥75%, or with more than 6 contractions per hour or both cervical changes and contractions as specified; or cervical dilation ≥8 cm with intact membranes) or; - there is evidence of non-reassuring fetal status or other clinical indication requiring immediate preterm delivery. - the obstetric care provider has a clinical suspicion or evidence of clinical chorioamnionitis or severe infection - she has received any systemic corticosteroid in the last two weeks (outside of trial); or - no prior ultrasound assessment of gestational age is available and an immediate ultrasound examination is not possible. - Other reasons include a major or lethal congenital fetal anomaly being identified, confirmed COVID infection deemed severe enough to require steroid treatment as per national standards of COVID treatment, is unwilling or unable to provide consent or assent (including due to active labour), or is currently participating in another clinical trial, or has previously participated in any ACTION trial or any other clinical indication where the treating clinician considers corticosteroids to be contraindicated. |
| --- |

**Table 1.** Site-specific sampling schedule for PK-PD study

| Site | Sample 1  Baseline (0-30 min pre first dose) | Sample 2  (1-4 hr post first dose) | Sample 3  (8-12 hr post-first dose) | Sample 4  (24-36 hr post-last dose) | Sample 5  (48-60 hr post-last dose) | Sample 6  (72-96 hr post last dose) | Birth samples for mother and cord |
| --- | --- | --- | --- | --- | --- | --- | --- |
| NIG-Ile Ife | Before first IMP dose | 1 | 8 | 24 | 48 | 72 | At birth |
| IND-Delhi | Before first IMP dose | 2 | 10 | 28 | 52 | 80 | At birth |
| NIG-Ibadan | Before first IMP dose | 3 | 11 | 32 | 56 | 88 | At birth |
| IND-Belagavi | Before first IMP dose | 4 | 12 | 36 | 60 | 96 | At birth |

*Windows for all draws ±30 minutes, except for sample 6.*

*Sample 6 – as close as possible to the stated time point for the site, otherwise at the point of discharge*
