# Supplementary material for: Population pharmacokinetics and pharmacodynamics of two dosing regimens of antenatal corticosteroids: protocol for a prospective nested study in a randomised controlled trial
Source: BMJ Open. 2025 Jun 8;15(6):e096523. doi: 10.1136/bmjopen-2024-096523 (PMC12161439; doi:10.1136/bmjopen-2024-096523)
Supplement: Supplementary file 2 [file bmjopen-2024-096523.reviewer_comments.pdf]

PEER REVIEW HISTORY

BMJ Open publishes all reviews undertaken for accepted manuscripts. Reviewers are asked to complete a checklist review form and are provided with free text boxes to elaborate on their assessment. These free text comments are reproduced below.

ARTICLE DETAILS

Title (Provisional)

Population pharmacokinetics and pharmacodynamics of two dosing regimens of antenatal corticosteroids: protocol for a prospective nested study in a randomized controlled trial.

Authors

The WHO ACTION-III PK PD Collaborators , The WHO ACTION-III PK PD Collaborators; Adejuyigbe, Ebunoluwa A.; OA, Adesina; Aripaka, Ramana Moorthy; Awoniyi, Francis Olubanji; Ayede, Adejumoke Idowu; BELLO, Nurudeen Ola; Bharti, Rekha; Dabral, Anjali; De Costa, Ayesha; Goudar, Shivaprasad S.; Gupta, Shuchita; Hasan , Zubair; ISAH, Dennis Anthony; Jusko, William; Kangle, Ranjit P; Krzyzanski, Wojciech; Lavin, Tina; Lawal, Olatunji Okikiola; Minckas, Nicole; Ogwu, Elijah-David Ukpahiu-Ojo; Oladapo, Olufemi Taiwo; Oyeniyi, Kabir Olatunde; Patil, Amaresh P; Pujar, Yeshita V; Ramachandran, Subramanian; Ranga, Sunil; Ray, Ezekiel Onoja; Sivasankaran, Suresh; Suri, Jyotsna; Somannavar, Manjunath S; Wadhwa, Nitya

---

VERSION 1 - REVIEW

**Reviewer**	**1**
**Name**	**Haas, David M.**
**Affiliation**	**Indiana University School of Medicine**
**Date**	**01-Jan-2025**
**COI**	**None**

This is a well written and important protocol. It capitalizes on an ongoing RCT ACTION-III, which will answer an important clinical question. PK and PD of antenatal corticosteroids are not well studied so this will be important.

1. My main issue is of clarification. It appears that all RCT participants will be recruited, whether they are receiving drug or not. And thus even participants on placebo will be having multiple blood draws. It may be helpful to clarify that the PK analysis will only be run on those who receive drug but that the PD analyses will be accomplished for all participants, including those who receive placebo.

2. Has there been consideration for collection of a specimen for genetic studies, in case there is a desire to look at pharmacogenetic influences on the concentrations or dynamics?

**Reviewer**	**2**
**Name**	**Babovic, Ivana**
**Affiliation**	**Clin Ctr Serbia**
**Date**	**09-Mar-2025**
**COI**	**None**

Dear Authors

Thanks for your interesting and well-documented submission. But I think that you lack one scientific information about PD and PK sterooid therapy They are related to metabolism of steroid in placenta and differences between two regimens of ACST as well as genetic differences between groups of patients.

VERSION 1 - AUTHOR RESPONSE

Editors/reviewer's comments	Authors response
**Reviewer 1:** Dr. David M. Haas, Indiana University School of Medicine	
This is a well written and important protocol. It capitalizes on an ongoing RCT ACTION-III, which will answer an important clinical question. PK and PD of antenatal corticosteroids are not well studied so this will be important.	Thank you for the insightful comment.
1. My main issue is of clarification. It appears that all RCT participants will be recruited, whether they are receiving drug or not. And thus even participants on placebo will be having multiple blood draws. It may be helpful to clarify that the PK analysis will only be run on those who receive drug but that the PD analyses will be accomplished for all participants, including those who receive placebo.	This PK-PD study is nested within a three-arm, double-blind trial, and the group allocation remains blinded. Therefore, PK and PD analyses will be conducted on all participants enrolled in the study, regardless of whether they belong to the active or placebo group. This approach will preserve blinding while also allowing for comparisons of pharmacodynamic parameters between women who receive the active drug versus the placebo.
2. Has there been consideration for collection of a specimen for genetic studies, in case there is a desire to look at pharmacogenetic influences on the concentrations or dynamics?	No samples for genetic studies were collected. This has been included as a limitation.

**Reviewer: 2**<br>**Dr. Ivana Babovic, Clin Ctr Serbia**	
Dear Authors<br>Thanks for your interesting and well-documented submission. But I think that you lack one scientific information about PD and PK steroid therapy They are related to metabolism of steroid in placenta and differences between two regimens of ACST as well as genetic differences between groups of patients.	You are right we have not considered these aspects in the present study. This has now been indicated as a limitation.
